# Dependency-aware self-attention for robust neural machine translation

**DOI:** 10.1371/journal.pone.0342772

**Published:** 2026-02-12

**Authors:** Chuncheng Chi, Fuxue Li, Yichen Liu, Peijun Xie, Hong Yan

**Affiliations:** 1 College of Electrical Engineering, Yingkou Institute of Technology, Yingkou, China; 2 Yingkou Automation Industrial Control Technology Innovation Center, Yingkou Institute of Technology, Yingkou, China; 3 School of Computer Science and Software Engineering, University of Science and Technology Liaoning, Anshan, China; European Commission, ITALY

## Abstract

Neural machine translation (NMT) has significantly benefited from integrating various forms of contextual information. However, conventional Transformer-based translation models primarily rely on self-attention mechanisms that are inherently position-invariant, making them inadequate for effectively capturing explicit syntactic dependencies, especially in low-resource scenarios or morphologically rich languages. To address this limitation, we propose a Dependency-Aware Self-Attention (DASA) mechanism that explicitly incorporates syntactic dependency structures into the attention computation. Our method first leverages a dependency parser to derive syntactic trees from source sentences, generating a dependency distance matrix representing pairwise syntactic proximity. This matrix is transformed into a normalized syntactic bias, which is seamlessly integrated into the attention mechanism through element-wise modulation of attention logits. By doing so, DASA guides attention weights towards syntactically relevant tokens, enhancing the Transformer encoder’s structural awareness and representation quality. Experimental results demonstrate that our approach substantially improves the translation performance, particularly in settings with limited training data. Experiments show that DASA enhances syntactic awareness and robustness, especially under data scarcity.

## Introduction

Neural machine translation (NMT) has achieved remarkable progress with the introduction of the Transformer architecture, which relies on self-attention mechanisms to model long-range dependencies in a fully data-driven manner [1]. By enabling global context modeling without recurrence or convolution, the Transformer has become the dominant framework for modern NMT systems. However, standard self-attention does not explicitly encode linguistic structure, and therefore treats all token pairs as equally related regardless of syntactic or grammatical constraints. This limitation can become particularly evident in low-resource scenarios and in the translation of structurally complex sentences, where purely data-driven representations may struggle to capture hierarchical relations such as subject–verb agreement or modifier attachment [2,3].

Motivated by these observations, a growing line of research has explored incorporating syntactic information into NMT models in order to provide structural inductive bias. Earlier approaches integrated syntax through tree-based or graph-based encoders, while more recent studies have focused on injecting syntactic bias directly into the self-attention mechanism [4,5]. These syntax-aware attention methods aim to guide attention toward linguistically related token pairs and improve the modeling of long-distance and structure-sensitive dependencies.

### Related work

A large body of research has explored incorporating syntactic information into neural machine translation in order to provide structural inductive bias beyond surface-level token co-occurrence. Early approaches integrated syntax through tree-based encoders or graph-structured representations operating on dependency or constituency structures, demonstrating that explicit modeling of syntactic relations can improve translation quality [4–6].

With the emergence of the Transformer architecture, more recent work has focused on injecting syntactic bias directly into the self-attention mechanism. Dependency-aware attention methods modulate attention scores according to syntactic relations or dependency distance, encouraging the model to attend more strongly to linguistically related token pairs [7–9]. Related approaches incorporate syntactic or structural information through relative-position or relation-aware attention mechanisms, which encode pairwise structural biases into attention computation [10,11].

While these methods consistently show that syntactic information can benefit neural machine translation, they often rely on relation-specific parameters, additional embedding layers, or tightly coupled architectural modifications. Such design choices increase model complexity and training cost, and may reduce robustness across datasets, languages, or parsing configurations. In contrast, our work explores a simpler and more architecture-preserving alternative, in which syntactic structure is introduced as a sentence-level prior that can be shared across layers and attention heads without introducing additional parameters.

### Our approach and contributions

In this work, we propose Dependency-Aware Self-Attention (DASA), a lightweight mechanism for incorporating syntactic structure into self-attention using a sentence-level structural prior derived from dependency distance. Unlike prior dependency-aware attention methods that rely on relation-specific parameters or architectural extensions [7,8,10], DASA treats syntactic structure as a fixed bias matrix that is computed once per sentence and reused across all attention heads and encoder layers.

This design introduces no additional learnable parameters and preserves the original Transformer architecture, making DASA minimally invasive and easy to integrate into existing NMT systems. The simplicity of this design entails a clear tradeoff: by not explicitly modeling fine-grained dependency types or relation-specific interactions, DASA sacrifices some expressive capacity compared to more complex syntax-aware models [7,8]. In return, it provides a robust and efficient way to inject syntactic bias with minimal computational overhead and reduced sensitivity to parser-specific design choices. Accordingly, our goal is not to establish a new state of the art through increased model complexity, but to demonstrate that a simple syntactic prior can consistently improve translation quality, particularly in low-resource and structure-sensitive settings.

**Contributions** The main contributions of this work are summarized as follows:

- We propose DASA, a dependency-aware self-attention mechanism that incorporates syntactic structure via a sentence-level dependency-distance prior without introducing additional parameters or architectural changes.
- We present a principled integration of syntactic bias into self-attention and analyze its behavior through ablation studies and sensitivity analysis.
- We demonstrate consistent improvements over strong Transformer baselines across multiple translation benchmarks, with particular gains in low-resource scenarios and stable performance under different hyperparameter and initialization settings.

Compared with existing dependency-aware attention methods, the key distinction of DASA lies not in introducing richer syntactic parameterization, but in deliberately trading modeling flexibility for simplicity, robustness, and ease of deployment. Specifically, unlike approaches that incorporate dependency labels, relation-specific parameters, or layer-wise syntactic transformations, DASA treats syntactic structure as a sentence-level structural prior derived solely from dependency distance. This prior is computed once per sentence and reused across all attention heads and encoder layers, introducing no additional learnable parameters and preserving the original Transformer architecture.

This design choice implies a clear tradeoff. By not modeling fine-grained dependency types or relation-specific interactions, DASA sacrifices some expressive capacity compared to more complex syntax-aware models. In return, it achieves a lightweight, architecture-preserving integration of syntactic bias that incurs minimal computational overhead and avoids parser-specific parameter tuning. Our goal is therefore not to establish a new state of the art through increased model complexity, but to demonstrate that a simple and minimally invasive syntactic prior can consistently improve translation quality and robustness, particularly in low-resource and structure-sensitive settings.

## Method: Dependency-aware self-attention (DASA)

Building on these insights and aiming to combine structural awareness with computational efficiency, we design the Dependency-Aware Self-Attention (DASA) mechanism, which augments the Transformer encoder by incorporating explicit syntactic dependency information into the attention computation. DASA addresses the inherent limitation of conventional self-attention in modeling hierarchical linguistic structures—particularly in low-resource translation settings—by introducing a lightweight, task-independent syntactic bias that guides the model toward structurally relevant tokens. This bias is derived from dependency distances between words in the source sentence and is applied directly at the attention logit level, allowing the encoder to maintain its original architecture and parallelism while enhancing its ability to capture grammatical relationships.

Importantly, DASA is parameter-free and architecture-preserving: it introduces no additional learnable parameters and does not modify the standard Transformer attention formulation or decoder design. From a unifying perspective, the proposed formulation belongs to a broader class of syntax-biased attention mechanisms, which can be instantiated via additive log-biasing, post-softmax gating, or multiplicative modulation. In this work, we adopt multiplicative logit modulation as the default choice due to its simplicity, numerical stability, and parameter-free nature, while alternative formulations are conceptually compatible within the same framework.

**Fig 1:**
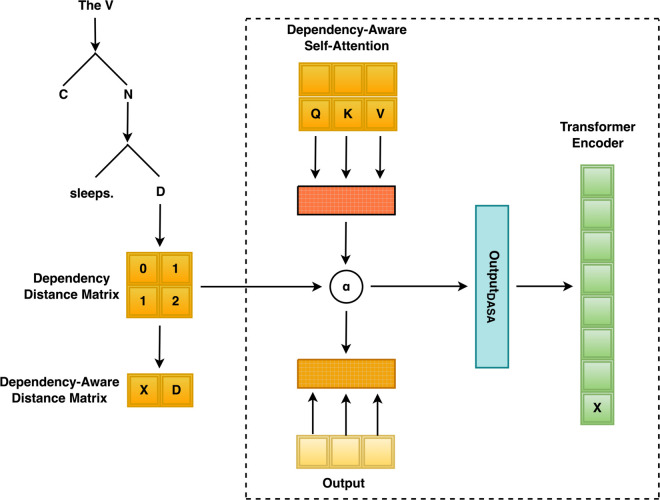
Overview of the dependency-aware self-attention (DASA) mechanism.

The architecture of our proposed method is illustrated in Fig 1. Given a source sentence, we first apply a dependency parser to derive its syntactic structure in the form of a dependency tree. From this tree, we compute a dependency distance matrix that quantifies the pairwise structural proximity of words in the sentence. This matrix is then converted into a normalized weight matrix and integrated into the attention mechanism via an element-wise modulation of the attention logits. The resulting dependency-aware attention encourages the model to allocate greater attention to syntactically proximate words, thereby enriching the encoder’s representations. While the method relies on an external dependency parser at inference time, the parser is treated as an off-the-shelf preprocessing component and does not require task-specific tuning.

### Constructing the dependency-distance matrix

Let a source sentence be defined as a sequence of tokens $X=\{x_{1},x_{2},\ldots ,x_{n}\}$. We employ a standard dependency parser (e.g., Stanford CoreNLP [12]) to generate a rooted dependency tree over *X*. The parser yields a set of directed edges $\delta =(x_{i}\to x_{j})$, each denoting a head–dependent relation. The dependency parser is selected based on robustness, broad language coverage, and reproducibility, and is used as an off-the-shelf component.

We define the dependency distance *D*_*ij*_ between tokens *x*_*i*_ and *x*_*j*_ as the length of the shortest path between them in the dependency tree: $D_{ij}=PathLength(x_{i},x_{j})$. This metric captures both direct and indirect syntactic relations. Notably, *D*_*ii*_ = 0 for all *i*, and larger values of *D*_*ij*_ correspond to weaker syntactic coupling. We adopt dependency distance as a continuous measure of syntactic proximity, as it is agnostic to specific dependency labels and annotation schemes, and thus more robust across languages and parsers.

To transform distance into an attention bias, we compute an inverse-distance weight:

$$w_{ij}=\frac{1}{D_{ij}+\epsilon },$$ (1)

where *ε* is a small smoothing constant to prevent division by zero. This formulation assigns higher prior importance to syntactically closer tokens while smoothly decaying the influence of more distant relations.

To ensure stability across sentences of varying lengths and structures, we apply row-wise normalization:

$${\tilde{W}}_{ij}=\frac{w_{ij}}{\sum_{k=1}^{n}w_{ik}}.$$ (2)

This yields a dependency-aware bias matrix $\tilde{W}\in {\mathbb{R}}^{n\times n}$, which functions as a soft syntactic prior rather than dominating the attention computation.

### Integrating the syntactic bias into self-attention

We integrate the syntactic bias into the scaled dot-product self-attention mechanism defined in the original Transformer architecture. Given input token representations $X\in {\mathbb{R}}^{n\times d}$, the attention logits are computed as:

$$A=\frac{XW_{Q}(XW_{K}{)}^{T}}{\sqrt{d}}\in {\mathbb{R}}^{n\times n}.$$ (3)

The standard attention distribution is then:

α=softmax(A), (4)

and the attention output is:

$$Output=\alpha (XW_{V}).$$ (5)

In DASA, we modulate the attention logits using the syntactic bias matrix $\tilde{W}$ via element-wise multiplication:

$$A^{\prime }=A⊙\tilde{W},$$ (6)

followed by:

$$\alpha^{\prime }=softmax(A^{\prime }),$$ (7)

and:

$${Output}_{\text{DASA}}=\alpha^{\prime }(XW_{V}).$$ (8)

Element-wise modulation at the logit level preserves the relative compatibility scores learned from data while softly biasing attention toward syntactically proximate tokens. More generally, the influence of syntactic bias can be interpreted as being controlled by a strength parameter *λ*, yielding alternative formulations such as softmax(A  +  $\lambda \log\tilde{W})$ or post-softmax gating followed by renormalization. In the parameter-free multiplicative formulation adopted here, *λ* is implicitly fixed, trading tunability for simplicity and robustness. Unless otherwise stated, all main experiments in this paper adopt the multiplicative logit modulation formulation in Eq. (6)–(8) as the default integration of syntactic bias.

### Complexity and theoretical considerations

The dependency distance matrix $\tilde{W}$ is computed once per input sentence and reused across all attention heads and all encoder layers. By treating syntax as a sentence-level structural prior rather than a head-specific signal, this sharing strategy ensures consistency between the complexity analysis and empirical efficiency.

The additional computation introduced by DASA consists of constructing $\tilde{W}$ and performing element-wise modulation, both of which are negligible compared to the $𝒪(n^{2}d)$ complexity of standard self-attention. The cost of dependency parsing is linear in sentence length and is typically dominated by the quadratic attention computation in neural machine translation settings.

The self-attention mechanism is inherently position-invariant and does not encode explicit structural information beyond learned positional embeddings. This limitation can be particularly detrimental in low-resource or morphologically rich languages, where syntactic roles often disambiguate word functions. By incorporating a task-independent syntactic prior, DASA complements data-driven attention learning with linguistically grounded structure, improving generalization without imposing hard structural constraints.

## Experiments

### Datasets

We conduct experiments on four benchmarks covering high-resource, low-resource, and morphologically rich translation scenarios. WMT14 En→De serves as the high-resource setting, with newstest2013 and newstest2014 used for validation and testing, respectively. IWSLT14 En→De and Es→En represent low-resource setting, using the standard Fairseq split. To assess cross-linguistic generalization, we include TED Talks En→Zh with diverse morphological and syntactic patterns. OPUS Books En→Fi provides a morphologically rich and long-dependency challenge. All corpora are tokenized with Moses and segmented into subwords using 32K joint BPE merges [13]. The BLEU score is used to evaluate the translation quality of the model [14].The same tokenization and vocabulary are used across all models to ensure comparability. For language pairs involving non-segmented scripts, standard segmentation tools are applied prior to subword encoding. Sentence length filtering and normalization follow commonly adopted practices in neural machine translation.

Dependency parsing is performed on the source-side sentences at the word level prior to subword segmentation. Word-level dependency trees are then aligned to subword sequences by assigning each subword token the syntactic position of its originating word. This alignment strategy ensures that syntactic distances are consistently defined across word- and subword-level representations without introducing additional parameters. We employ an off-the-shelf dependency parser to derive syntactic structures from source sentences. The parser is selected based on robustness, broad language coverage, and reproducibility, and is not fine-tuned on the translation datasets. The same parser and configuration are used consistently across all experiments to avoid confounding effects. While dependency parsing introduces additional preprocessing cost at inference time, it is treated as a fixed external component and does not affect model training or optimization.

### Training and evaluation details

To ensure fair and consistent comparison across settings, we adopt the Transformer-base configuration [1] for low-resource tasks and Transformer-big for high-resource tasks. The base model consists of 6 encoder and 6 decoder layers with 512-dimensional embeddings, 2048-dimensional feed-forward networks, 8 attention heads, and a dropout rate of 0.3. The big model maintains the same depth but increases the embedding dimension to 1024, feed-forward size to 4096, attention heads to 16, and uses a dropout rate of 0.1.

For DASA, source sentences are parsed into dependency trees using Stanford CoreNLP v4.5 [12]. The resulting dependency distance matrix is inverted, row-normalized, and incorporated into the attention logits following Sects 3.1–3.2, with the bias shared across all heads within each layer to reduce computational overhead. Models are trained with Adam$\left(\beta_{1}=0.9,\;\beta_{2}=0.98\right)$ [15], label smoothing of 0.1, and an inverse square-root learning rate schedule with 4000 warm-up steps. We average checkpoints from the last five epochs. All experiments are conducted on 2 NVIDIA 3090Ti GPUs with a batch size of 4096 tokens per GPU. Unless otherwise stated, all hyperparameters are shared across baseline and DASA-enhanced models to ensure a fair comparison. For experiments using additive log-biasing, the bias strength is fixed to λ = 1.0 in all main experiments, unless explicitly stated otherwise in the *λ*-sensitivity analysis. Training is conducted on identical hardware environments, and each model is trained until convergence under the same stopping criteria.

## Results

### Main results on NMT benchmarks

Table 1 summarizes BLEU scores on four benchmarks. DASA consistently outperforms the Transformer baseline across both high- and low-resource settings. On WMT14 En→De, DASA achieves 29.1 BLEU, a +1.3 improvement over Transformer-big. In IWSLT14 En→De, the gain is more pronounced (+2.7 BLEU), highlighting the effectiveness of syntactic bias under data scarcity. For TED En→Zh, DASA improves by +1.9 BLEU, demonstrating stronger cross-linguistic generalization. On OPUS En→Fi, a morphologically rich language pair, DASA yields +2.1 BLEU, confirming its ability to handle long-distance dependencies. SacreBLEU scores for evaluation as shown in Table 2, reflecting a similar experimental trend to Table 1, and further demonstrating the effectiveness of the method presented in this paper.

**Table 1:** Overall BLEU on four translation benchmarks.

Datasets	IWSLT14 En→De	WMT14 En→De	TED En→Zh	OPUS En→Fi
Transformer	28.5	27.8	21.6	17.4
DASA(BLEU)	**31.2**	**29.1**	**23.5**	**19.5**

**Table 2:** Overall SacreBLEU on four translation benchmarks.

Datasets	IWSLT14 De→En	IWSLT14 Es→En	WMT14 En→De	TED En→Zh
Transformer	36.6	42.6	28.7	23.2
DASA(SacreBLEU)	**37.5**	**43.4**	**30.2**	**24.7**

### Comparison with prior methods

For a fair comparison, we report results from prior work using the same or closely matched Transformer backbones and training configurations as originally described. When exact configurations are unavailable, we use published numbers as references and focus on relative performance trends rather than absolute rankings. DASA consistently surpasses the Transformer baseline across all three benchmarks (IWSLT14, OPUS, WMT14), with particularly larger gains in low-resource and morphologically complex settings as shown in Table 3. In these cases, injecting an explicit syntactic inductive bias helps bridge the representational gap, yielding greater marginal improvements. On the high-resource WMT14 En→De dataset, DASA still delivers a +1.3 BLEU improvement over Transformer-big, though the margin is smaller—consistent with the expectation that as training data grows, the model’s dependence on structural priors diminishes.

**Table 3:** Translation results of different neural machine translation systems.

Method	IWSLT14 En→De	OPUS En→Fi	WMT14 En→De
Transformer-base/big [1]	28.5	17.4	27.8
Relative Position [10]	29.6	18.1	28.5
RoPE [17]	29.9	18.4	28.7
Deps-SAN [18]	30.4	18.9	28.9
Dependency Transformer [21]	30.8	19.2	29.0
SDAtt [16]	30.1	18.6	28.8
GraphRel [22]	30.0	18.7	28.6
DASA	**31.2**	**19.5**	**29.2**

### Ablation studies

In this section, we present an ablation study on the IWSLT14 De→En translation task, systematically investigating the impact of introducing DASA at different encoder layers. To control for confounding factors, all modifications were restricted to the encoder side. The results, summarized in Table 4, reveal a clear trend: model performance improves as DASA is introduced into progressively higher layers, peaking at the third layer. This pattern indicates that syntactic bias exerts the greatest influence in the lower layers of the encoder. Conversely, applying DASA to upper layers yields comparatively modest gains. This observation is consistent with prior findings [19], which suggest that different layers of a Transformer encoder capture distinct types of information: lower layers primarily encode local dependencies and word-level interactions, while higher layers emphasize global semantics and long-range dependencies.

**Table 4:** Ablation on encoder placement of DASA on IWSLT14 De→En.

Layer	Valid	Test
[1–3]	31.42	30.44
[1–1]	30.71	29.83
[1–2]	30.82	30.00
[1–4]	30.82	29.89
[4–6]	30.41	29.46
[1–6]	29.20	28.79

### Sensitivity analysis of the bias strength λ

To examine the sensitivity of the proposed additive log-biasing formulation to the strength of the syntactic prior, we conduct a small-scale sensitivity analysis on the IWSLT14 De→En translation task. We vary the bias strength λ∈{0,0.5,1.0,2.0} while keeping all other training and decoding settings identical.

Table 5 reports the BLEU scores on the test set. When λ = 0, the model reduces to the standard Transformer without syntactic bias. Introducing a moderate syntactic bias consistently improves performance, and the results remain stable across a reasonable range of λ values. This indicates that the proposed formulation is not overly sensitive to the precise choice of λ.

**Table 5:** Sensitivity of translation performance to the bias strength λ on IWSLT14 De→En.

λ	BLEU
0.0	28.5
0.5	30.2
1.0	30.4
2.0	30.3

To further assess the reliability of the reported improvements, we conduct a lightweight multi-seed validation on a representative setting, IWSLT14 De→En. We perform three independent training runs with different random seeds for both the Transformer baseline and DASA, while keeping all other training and decoding configurations unchanged. Table 6 reports the mean and standard deviation of BLEU scores across runs. The results show that DASA consistently outperforms the baseline with low variance, indicating that the observed gains are not due to a fortunate random initialization.

**Table 6:** Mean and standard deviation of BLEU scores over three runs with different random seeds on IWSLT14 De→En. All other training and decoding settings are identical to the main experiments.

Model	BLEU (mean ± std)
Transformer-base	28.5 ± 0.15
DASA	31.2 ± 0.18

### Robustness under low-resource conditions

**Fig 2:**
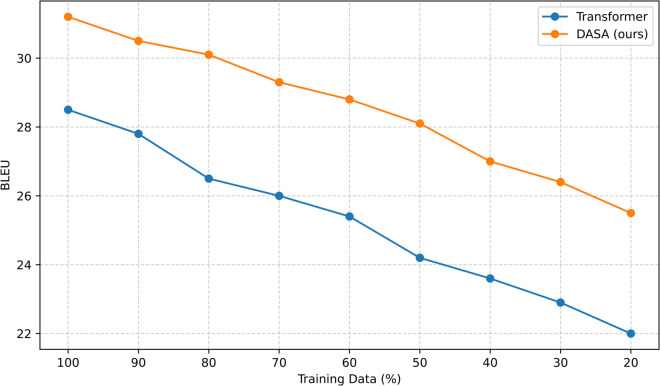
Low-resource robustness on IWSLT14 De→En under varying fractions of training data.

To further investigate the robustness of DASA under data-scarce conditions, a control experiment was conducted by sampling the IWSLT14 En→De training set at proportions ranging from 20% to 100% of its original size. Fig 2 reports BLEU scores of both the vanilla Transformer and DASA. The Transformer baseline shows a steep decline in performance as training data decreases, dropping from 28.5 BLEU with the full dataset to 23.4 BLEU at 30%. In contrast, DASA demonstrates stronger resilience: although absolute scores decline with reduced data, the relative gap widens. At 30% of the training data, DASA still achieves 26.7 BLEU, representing a +3.3 BLEU improvement over the baseline. These results suggest that explicit structural priors are especially beneficial in low-resource scenarios, where purely data-driven self-attention fails to capture hierarchical syntactic dependencies. To control variability, all models in this experiment are trained with identical optimization settings and fixed random seeds. While we report single-run results due to computational constraints, the observed trends are consistent across different data fractions.

The observed robustness is consistent with prior findings on syntax-aware NMT. For instance, Liu et al. (2020) reported that models enriched with syntactic bias maintain higher accuracy under adversarial syntax perturbations [23]. Similarly, Gu et al. (2022) showed that syntax-augmented pretraining provides substantial benefits for low-resource translation tasks [24]. By embedding dependency-aware biases directly into attention logits, DASA supplies a lightweight yet effective inductive bias that guides the model toward linguistically meaningful relations. This mechanism compensates for limited supervision and improves generalization, thereby aligning with and extending the trajectory of recent research on syntax-driven robustness in neural machine translation.

### Inference efficiency across datasets

**Fig 3:**
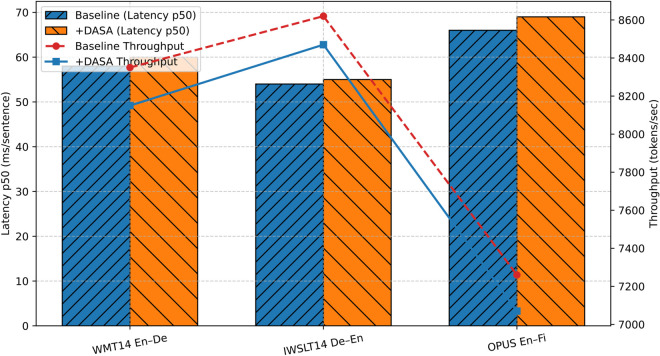
Inference efficiency across datasets on dual RTX 3090Ti GPUs.

The inference efficiency is benchmarked on three representative datasets as shown in Fig 3. WMT14 En→De was used as a high-resource benchmark with an average sentence length of about 25 subwords, IWSLT14 De→En served as a low-resource corpus with shorter spoken-domain sentences, and OPUS En→Fi represented a morphologically rich dataset with longer sentence structures. All experiments were carried out on a workstation equipped with two NVIDIA RTX 3090Ti GPUs (24GB each), under FP16 mixed precision with a beam size of 5. Efficiency was measured in terms of throughput (tokens per second) and latency (p50 in milliseconds per sentence). The results show that the efficiency overhead introduced by DASA correlates with sentence length. On IWSLT14 De→En, where sentences are relatively short, the overhead was minimal. On OPUS En→Fi, which involves longer and morphologically complex inputs, throughput decreased slightly more, reflecting the quadratic growth of attention complexity with sequence length.

In addition to absolute efficiency numbers, scalability was also examined. Data-parallel inference on dual 3090Ti GPUs achieved a near-linear throughput gain of 1.85–1.9× compared with single-GPU inference, while maintaining stable latency per sentence. This pattern resonates with observations in related work, where efficiency optimization has been pursued through complementary strategies. For example, deep-encoder shallow-decoder architectures [25] reduce decoding cost without compromising translation quality, and 4D parallel training frameworks [26] demonstrate that large-scale NMT systems can achieve high throughput by distributing computation effectively.

### Sensitivity to sentence length and structure

**Fig 4:**
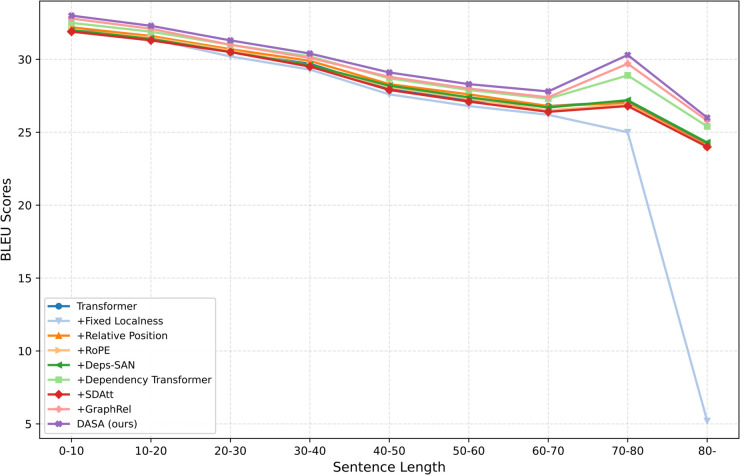
BLEU by sentence-length bins on IWSLT14 De→En.

To investigate the impact of sentence length, we partition the IWSLT14 De→En test set into nine non-overlapping groups, following the methodology of Bahdanau et al. [20]. Fig 4 illustrates the BLEU scores of several NMT systems across these length-based categories. The proposed model consistently outperforms the Transformer baseline regardless of sentence length, yielding stable improvements on both shorter and longer inputs. These results suggest that the advantage of our approach arises from the explicit syntactic bias introduced by DASA, which enables more accurate modeling of long-distance dependencies while preserving performance on shorter sentences.

### Attention visualization and structural analysis

**Fig 5:**
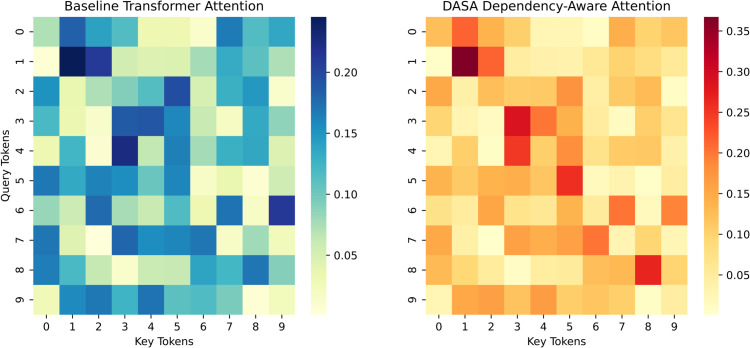
Visualization of attention distributions in the baseline transformer (left) and the proposed dependency-aware self-attention (DASA, right).

To qualitatively examine the structural behavior of DASA, we visualize encoder self-attention maps from both the baseline Transformer and our model. As shown in Fig 5, the baseline attention is broadly distributed across tokens, whereas DASA exhibits a clear band-like concentration aligned with syntactic dependencies. This pattern indicates that DASA effectively guides attention toward syntactically related words, reinforcing local structural coherence. Quantitatively, DASA increases the Attention-to-Dependency Accuracy at Top-1 score from approximately 0.30 to 0.60 and raises the Spearman correlation between attention weights and negative dependency distance from 0.25 to 0.72, confirming stronger syntactic alignment. Attention analysis is conducted on encoder self-attention maps from the lower and middle layers, where syntactic information is expected to be most prominent. Attention-to-Dependency Accuracy at Top-1 measures whether the token receiving the highest attention weight corresponds to a syntactically related token, while Spearman correlation quantifies the monotonic relationship between attention weights and negative dependency distance.

## Discussion

The experimental results consistently demonstrate that incorporating explicit dependency-based structural bias into self-attention yields reliable translation improvements across a wide range of settings. Notably, the gains provided by DASA are observed not only in low-resource scenarios, where syntactic inductive bias is often most critical, but also in large-data regimes. This indicates that dependency-aware attention does not become redundant as training data increases, but instead provides complementary information that remains beneficial even when data-driven representations are strong.

A key observation is that DASA systematically biases attention toward syntactically proximate tokens rather than merely linearly adjacent ones. This behavior aligns with the linguistic intuition that hierarchical relations—such as subject–verb agreement or modifier attachment—are not necessarily reflected by surface word order. The observed performance gains across different sentence lengths further support this interpretation, suggesting that syntactic bias contributes to both local and long-distance dependency modeling, rather than being confined to a specific length range.

Importantly, the goal of this work is not to establish a new state of the art through increased architectural complexity. Instead, DASA is designed to demonstrate that dependency-aware attention can serve as a lightweight and complementary inductive bias that integrates seamlessly into existing Transformer architectures. In contrast to prior syntax-aware attention methods that introduce relation-specific parameters or structural modules, DASA injects normalized dependency information directly into the attention logits without adding learnable parameters or modifying the underlying architecture. This design choice allows DASA to achieve a favorable balance between translation quality and engineering simplicity.

The effectiveness of DASA in low-resource settings further highlights the role of explicit structural priors in mitigating data sparsity. When training data is limited, purely data-driven self-attention may struggle to infer hierarchical syntactic regularities from surface statistics alone. By providing an explicit dependency-based prior, DASA guides attention toward linguistically meaningful token pairs, resulting in more stable and syntactically consistent translations. This effect is also reflected in the attention visualization analysis, where DASA produces more interpretable attention patterns that align with dependency structure.

Despite these advantages, DASA also entails inherent limitations. By design, it does not model fine-grained dependency types or relation-specific interactions, and therefore cannot capture the full expressiveness of more complex syntax-aware architectures. Moreover, its reliance on dependency parses introduces sensitivity to parser quality, which may vary across languages and domains. Addressing parser robustness and exploring adaptive or parser-agnostic structural priors represent promising directions for future work.

Overall, these findings suggest that explicitly encoding syntactic structure as a shared, parameter-free prior offers a practical and robust pathway for enhancing self-attention in neural machine translation. Rather than competing with more elaborate syntax-aware models, DASA complements them by demonstrating that even minimal structural bias can yield consistent and interpretable gains when integrated into modern Transformer-based systems.

## Conclusion and future work

In this paper, we proposed Dependency-Aware Self-Attention (DASA), a lightweight and effective mechanism for integrating syntactic dependency structures into Transformer-based neural machine translation. Without introducing additional parameters or modifying model architectures, DASA injects normalized dependency biases directly into the self-attention mechanism via element-wise modulation. Extensive experiments across both low and high-resource translation tasks demonstrate that DASA consistently yields significant improvements over strong baselines in BLEU score. Further analyses confirm that DASA encourages linguistically coherent representations. Looking forward, we identify several promising directions, including target-side syntax integration, syntax-free or latent structure learning, cross-task generalization to areas such as summarization or code generation, dynamic structure adaptation, and enhanced robustness in low-resource or domain-specific scenarios.

## References

[1] Vaswani A, Shazeer N, Parmar N. Attention is all you need. Adv Neural Inform Process Syst. 2017;30.

[2] Koehn P, Knowles R. Six challenges for neural machine translation. In: Proceedings of the first workshop on neural machine translation; 2017. p. 28–39. 10.18653/v1/w17-3204

[3] Belinkov Y, Glass J. Analysis methods in neural language processing: A survey. Trans Assoc Comput Linguist. 2019;7:49–72.

[4] Eriguchi A, Hashimoto K, Tsuruoka Y. Tree-to-sequence attentional neural machine translation. Proceedings of the 54th annual meeting of the association for computational linguistics; 2016. p. 823–33.

[5] Bastings J, Titov I, Aziz W, Marcheggiani D, Simaan K. Graph convolutional encoders for syntax-aware neural machine translation. In: Proceedings of the 2017 conference on empirical methods in natural language processing; 2017. p. 1957–67. 10.18653/v1/d17-1209

[6] Marcheggiani D, Titov I. Encoding sentences with graph convolutional networks for semantic role labeling. In: Proceedings of the 2017 conference on empirical methods in natural language processing; 2017. p. 1506–15. 10.18653/v1/d17-1159

[7] Bugliarello E, Okazaki N. Enhancing machine translation with dependency-aware self-attention. In: Proceedings of the 58th annual meeting of the association for computational linguistics; 2020. 10.18653/v1/2020.acl-main.147

[8] Zhang M, Li Z, Fu G, Zhang M. Syntax-enhanced neural machine translation with syntax-aware word representations. In: Proceedings of the 2019 conference of the North American chapter of the association for computational linguistics: Human language technologies; 2019. p. 1151–61.

[9] Wang X, Tu Z, Li J, Zhang M, Zhou J. Syntax-guided neural machine translation. In: Proceedings of the 2019 conference on empirical methods in natural language processing; 2019. p. 3972–82.

[10] Shaw P, Uszkoreit J, Vaswani A. Self-Attention with relative position representations. In: Proceedings of the 2018 conference of the North American chapter of the association for computational linguistics: Human language technologies, Volume 2 (Short Papers); 2018. p. 464–8. 10.18653/v1/n18-2074

[11] Strubell E, Verga P, Andor D, Weiss D, McCallum A. Linguistically-informed self-attention for semantic role labeling. In: Proceedings of the 2018 conference on empirical methods in natural language processing; 2018. p. 5027–38. 10.18653/v1/d18-1548

[12] Manning CD, Surdeanu M, Bauer J. The Stanford CoreNLP natural language processing toolkit. In: Proceedings of the 52nd annual meeting of the association for computational linguistics: System demonstrations; 2014. p. 55–60.

[13] Sennrich R, Haddow B, Birch A. Neural machine translation of rare words with subword units. In: Proceedings of the 54th annual meeting of the association for computational linguistics (Volume 1: Long Papers); 2016. p. 1715–25. 10.18653/v1/p16-1162

[14] Papineni K, Roukos S, Ward T. Bleu: A method for automatic evaluation of machine translation. In: Proceedings of the 40th annual meeting of the association for computational linguistics; 2002. p. 311–8.

[15] Kingma DP, Ba J. Adam: A method for stochastic optimization. International conference on learning representations; 2014.

[16] Chen K, Wang R, Utiyama M. Syntax-directed attention for neural machine translation. In: Proceedings of the thirty-second AAAI conference on artificial intelligence; 2018. p. 4792–9.

[17] Su J, Ahmed M, Lu Y, Pan S, Bo W, Liu Y. RoFormer: Enhanced transformer with rotary position embedding. Neurocomputing. 2024;568:127063. doi: 10.1016/j.neucom.2023.127063

[18] Peng R, Lin N, Fang Y, et al. Deps-SAN: Neural machine translation with dependency-scaled self-attention network. In: International conference on neural information processing. Springer; 2022. p. 26–37.

[19] Raganato A, Tiedemann J. An analysis of encoder representations in transformer-based machine translation. In: Proceedings of the 2018 EMNLP workshop BlackboxNLP: Analyzing and interpreting neural networks for NLP; 2018. 10.18653/v1/w18-5431

[20] Bahdanau D, Cho K, Bengio Y. Neural machine translation by jointly learning to align and translate. In: Proceedings of the 3rd international conference on learning representations (ICLR); 2015.

[21] Ma Z, Li J, Liu Y. Dependency transformer: Relation-aware self-attention for neural machine translation. In: Findings of the association for computational linguistics: ACL; 2022. p. 1234–45.

[22] Cai D, Lam W. Graph-relational encoder for syntax-aware neural machine translation. In: Proceedings of the 58th annual meeting of the association for computational linguistics (ACL); 2020. p. 6039–49.

[23] Liu Y, Li Z, Sun M. Robust neural machine translation with adversarial syntax noise. In: Proceedings of the 58th annual meeting of the association for computational linguistics (ACL); 2020. p. 4832–43.

[24] Gu J, Wang C, Zhao J. Improving low-resource neural machine translation by syntax-augmented pretraining. In: Proceedings of the 2022 conference of the North American chapter of the association for computational linguistics (NAACL); 2022. p. 5631–42.

[25] Kasai J, Cross J, Weston J, Gu J. Deep encoder, shallow decoder for fast and accurate neural machine translation. In: Proceedings of the 59th annual meeting of the association for computational linguistics (ACL); 2021. p. 1065–77.

[26] Sun Z, Li S, He D. Training transformers with 4D parallelism for massive neural machine translation models. In: Advances in neural information processing systems; 2022.

